# Understanding reasons for treatment interruption amongst patients on antiretroviral therapy – A qualitative study at the Lighthouse Clinic, Lilongwe, Malawi

**DOI:** 10.3402/gha.v7.24795

**Published:** 2014-09-30

**Authors:** Julia Tabatabai, Ireen Namakhoma, Hannock Tweya, Sam Phiri, Paul Schnitzler, Florian Neuhann

**Affiliations:** 1 Institute of Public Health, University of Heidelberg, Heidelberg, Germany; 2 Department of Infectious Diseases, Virology, University of Heidelberg, Heidelberg, Germany; 3 Reach Trust, Lilongwe, Malawi; 4 The Lighthouse Trust, Lilongwe, Malawi; 5 The International Union against Tuberculosis and Lung Diseases, Paris, France

**Keywords:** treatment interruption, antiretroviral therapy, adherence, retention in care, Malawi

## Abstract

**Background:**

In recent years, scaling up of antiretroviral therapy (ART) in resource-limited settings moved impressively towards universal access. Along with these achievements, public health HIV programs are facing a number of challenges including the support of patients on lifelong therapy and the prevention of temporary/permanent loss of patients in care. Understanding reasons for treatment interruption (TI) can inform strategies for improving drug adherence and retention in care.

**Objective:**

To evaluate key characteristics of patients resuming ART after TI at the Lighthouse Clinic in Lilongwe, Malawi, and to identify their reasons for interrupting ART.

**Design:**

This study uses a mixed methods design to evaluate patients resuming ART after TI. We analysed an assessment form for patients with TI using pre-defined categories and a comments field to identify frequently stated reasons for TI. Additionally, we conducted 26 in-depth interviews to deepen our understanding of common reasons for TI. In-depth interviews also included the patients’ knowledge about ART and presence of social support systems. Qualitative data analysis was based on a thematic framework approach.

**Results:**

A total of 347 patients (58.2% female, average age 35.1±11.3 years) with TI were identified. Despite the presence of social support and sufficient knowledge of possible consequences of TI, all patients experienced situations that resulted in TI. Analysis of in-depth interviews led to new and distinct categories for TI. The most common reason for TI was travel (54.5%, *n*=80/147), which further differentiated into work- or family-related travel. Patients also stated transport costs and health-care-provider-related reasons, which included perceived/enacted discrimination by health care workers. Other drivers of TI were treatment fatigue/forgetfulness, the patients’ health status, adverse drug effects, pregnancy/delivery, religious belief or perceived/enacted stigma.

**Conclusions:**

To adequately address patients’ needs on a lifelong therapy, adherence-counselling sessions require provision of problem-solving strategies for common barriers to continuous care.

In recent years, scaling up of public health programmes in resource-limited settings made considerable progress towards universal access to antiretroviral therapy (ART) (1). By the end of 2011, an estimated 8 million patients in low- and middle-income countries, equalling 54% of those in need, received ART (2). Expanding coverage has been associated with a significant decrease in morbidity and mortality related to the human immunodeficiency virus (HIV) (3–7), and in reducing potentially the risk of HIV transmission (8). However, the effectiveness of ART is determined by continuous access to treatment and retention in care (9). As demonstrated by a systematic review of sub-Saharan countries, one in four patients does not remain in care 2 years after initiation of therapy (10).

The World Health Organization (WHO) defines adherence broadly as ‘the extent to which a person's behaviour – taking medication, following a diet, and/or executing lifestyle changes – corresponds with agreed recommendations from a health care provider’ (11). Rosen et al. further differentiate between day-to-day drug adherence, meaning the mere daily intake of medication as prescribed, and long-term retention in care (12). The latter puts adherence into a broader context embracing lifelong adherence to medication but also continuous attendance of programme activities such as regular clinical visits, counselling and patient education. In the context of increasing numbers of patients in care, the continuous lifelong treatment at the end of the HIV care pathway is of growing importance (13). The patients’ engagement in care and the prevention of permanent or temporary losses is crucial for the effectiveness and success of ART, particularly for future test-and-treat strategies for HIV prevention (14). The probability of sexual HIV transmission is linked to viral load levels (15), and effective ART has been shown to reduce further transmission of HIV and consequently the HIV incidence (16).

Long-term retention in care and day-to-day drug adherence can prevent poor clinical outcomes for the individual patient as well as the development and spread of drug resistance mutations within a population (17–20). Even though developing countries achieve similar or even higher adherence rates compared to those observed in developed countries (21–25), their ART regimen options and technical ability to detect drug resistance are limited. Under these circumstances, the preventive value of adherence becomes even more pronounced as it may at least partially compensate such deficits.

Non-adherence can either be expressed as percentage of missed doses or as frequency and duration of complete treatment interruption (TI) (26). TI has been associated with a higher risk of drug resistance than intermittent missed doses (20, 27). Consequently, it may be a more useful predictor of clinical outcomes than pill count alone (28).

A recent study from rural Malawi indicated that after initiation of ART, retention in care shows a sharp drop during the first months, primarily caused by high mortality rates. In contrast, long-term retention is subject to a more gradual decline mainly due to lost-to-follow-up (LTFU) (29). Tweya et al. showed that patients LTFU are a heterogeneous group and include patients who died, received ART from another clinic or other source, or actually stopped therapy (30). However, a considerable proportion of patients stopping ART resume therapy after some time, which is defined as TI (30, 31). This is accompanied by a high risk of treatment failure caused by evolving drug resistance (32).

Known reasons for TI include a range of economic, structural, social and behavioural barriers to continuous care (23). Approaches to increase the understanding of factors associated with TI often rely on clinical data about patient demographics and treatment regimen. Although this information can be easily obtained and is useful in defining risk groups, qualitative research can provide a deeper understanding of patients’ individual barriers to continuous care (33). In the past, treatment costs were frequently reported to represent a major obstacle, but this changed after ART became free of charge in most sub-Saharan countries. Instead, more recent research highlights transport costs as a common impediment (34). In addition, factors related to the patient–health care provider relationship must not be overlooked (22). Behavioural factors such as intrinsic motivation and knowledge about HIV and ART are interlinked with acceptance of medication and consequently adherence to therapy (35). Consequently, research efforts should aim at a more profound understanding of reasons for TI to assure the integration of needs-based adherence support into programme design. This study aimed at understanding TI in greater depth by exploring the following research questions:

1. What are the key characteristics of patients with confirmed TI?
2. Which factors do patients have at their disposal to support their day-to-day drug adherence and retention in care?
3. What main reasons do these patients report for interrupting ART?

## Methods

### Study setting

This study was conducted at the Lighthouse Clinic in Lilongwe, Malawi, one of the largest centres for ART in the country. Since 2004, ART and regular clinical reviews are provided free of charge and in accordance with the national scale up programme and WHO's *Universal Access* strategy (19, 36, 37). Lighthouse routinely uses a real-time electronic data system (EDS) for the collection of patient data and documentation of all visits, prescriptions and dispensing. During clinic visits, patients’ adherence is assessed via pill count and patients for whom the EDS calculates an adherence level <95% are referred to one to one adherence-counselling sessions. More details about the HIV epidemic in Malawi, the national ART programme as well as the Lighthouse as a centre of excellence for ART provision can be found elsewhere (30, 36–43).

#### The Back-to-Care Programme

In 2006, the *Back-to-Care Programme* (B2C) was initiated to improve patient retention in care via early active follow-up. The programme uses the EDS to identify patients who are overdue for their scheduled appointment ≥21 days, defined as patients LTFU (30, 32). These patients or their relatives/treatment supporters are contacted via mobile phone and/or home visits in order to determine their actual ART status. Using the standardized B2C form, the team documents information about the patient's survival status, the last clinic visit, the history since the last visit as well as future intentions to continue ART and return to care. Questions about the history since the last visit include possible alternate ART sources, the mode and frequency of pill intake and also possible reasons for stopping ART. The B2C form mainly consists of a closed format with either multiple options, date specifications or dichotomous options (yes/no). Free-text commentary fields allow for further specifications. The ART status of the patient is recorded as either *unsuccessful tracing attempt, death of the patient, transfer to another ART facility*, *uninterrupted therapy with alternate ART source*, *never started ART* or *stop of all prescribed antiretroviral drugs*. All patients found alive who did not transfer to another ART facility are encouraged to return to the Lighthouse to resume therapy (30).

### Study population

**Fig. 1:**
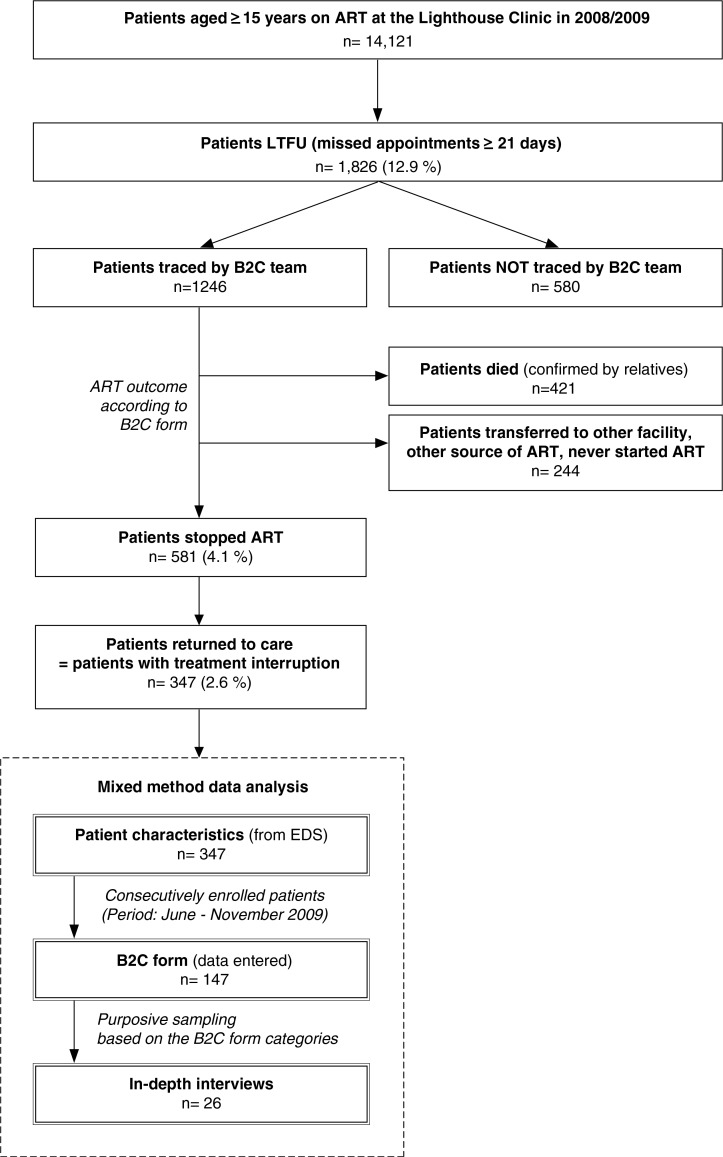
Study population. Eligibility criteria were ≥15 years, documented treatment interruption of ≥21 days (between January 2008 and November 2009), resumption of ART. In total, patient characteristics of 347 patients were analysed, 147 B2C forms were entered into an MSAccess database and 26 in-depth interviews were conducted for descriptive analysis and to gain a further understanding of reasons for treatment interruption. ART: Antiretroviral therapy; B2C: Back-to-Care; EDS: Electronic data system; LTFU: Lost-to-Follow up

This study included patients ≥15 years of age who resumed ART after documented TI between January 2008 and November 2009. TI was given if the ART status assessed by the B2C programme was ‘stop of all prescribed antiretroviral drugs’ and if these patients returned to clinic for ART resumption within the study period between June and November 2009 (Fig. 1). Duration of TI was calculated as the difference between estimated last pill day and the day of return to the clinic.

### Data collection

This study used a mixed methods analysis of quantitative and qualitative data to evaluate patients resuming ART after TI: their key characteristics, factors supporting adherence and retention in care, as well as reasons for stopping ART (Fig. 1). We quantified reasons for TI using the B2C forms with pre-defined categories. In addition, we performed in-depth interviews and evaluated free-text comments of the B2C forms to 1) identify additional reasons for TI and 2) gain a better understanding of the individual circumstances leading to TI.

#### Patients’ characteristics

For all patients resuming therapy after TI (*n*=347), routinely collected patient data from the EDS (demographics, ART regimen, duration and frequency of TI) was transferred into an MSAccess database.

#### B2C form

First of all, we compiled existing paper-based interview forms from the B2C programme of patients with confirmed TI. The information captured in the B2C forms is filled in by the B2C team based on answers obtained via phone interviews or personal contact in the field before these patients decide to return to care for resumption of ART. In these forms, the B2C team summarizes the patient's reasons for stopping ART by selecting one or more of eight pre-defined categories: 1) treatment fatigue/forgetfulness, 2) adverse drug effects, 3) weakness/sickness, 4) religious reasons, 5) travel, 6) traditional medicine, 7) transport costs or 8) other reason. The latter used an open question field to allow for further specification or to record not-listed reasons. The pre-defined categories were developed on the basis of common reasons for TI as found in the literature when the B2C programme was initiated in 2006 (30).

We retrospectively analysed a subset of 147 forms of all patients with TI who appeared in person at the clinic within the enrolment period June to November 2009. This enrolment strategy ensured that these patients could also be approached for a prospective re-evaluation of their reasons for TI (in-depth interview). These patients were also consecutively enrolled in a linked study evaluating virological outcomes in patients with TI (32). The paper-based forms were double entered into a database for further analysis.

#### In-depth interviews

Between June and November 2009, a subset of the enrolled B2C patients (*n*=26/147) were approached on site for semi-structured in-depth interviews asking about individual circumstances that led to TI. Reasons for TI derived from the B2C form were used to select key topics for the interview guide. We applied purposive sampling based on the pre-defined B2C form categories to assure selection of typical cases and to re-evaluate these categories. Patients were prospectively recruited until a point of saturation was reached, meaning that no new aspects or further themes emerged (44). Evaluating factors generally known to support drug adherence and retention in care, respondents’ perception of ART, knowledge about the risk of treatment failure, the importance of adherence, and the presence of support structures were also assessed. In-depth interviews were conducted in the local language Chichewa by one trained, experienced and independent interviewer (not employed at Lighthouse). Interviews were conducted in clinic rooms to ensure privacy and an undisturbed environment, preceded in a conversational style and were tape-recorded.

### Data analysis

Quantitative data from the EDS and the B2C form was analysed using STATA^®^/IC11 (StataCorp. LP, College Station, TX). Continuous variables were described by their means and standard deviation for normally distributed data. Descriptive statistics also included ranges and proportions, as appropriate. Significance of difference was tested using Students *t* test and *χ* ^2^ as appropriate. Regression analysis with TI/duration of TI as dependent variable adjusted for sex and/or age was performed to evaluate key characteristics of patients with TI. Level of significance was set at *p*≤0.05 and 95% confidence interval was used throughout.

Qualitative data of the B2C form was independently analysed and discussed by two researchers. Comments specifying one of the pre-defined categories were used to transform these into subcategories, and new upcoming categories were added as they emerged from the data.

Qualitative analysis of in-depth interviews was performed using a thematic framework approach (45). First, interviews were transcribed verbatim and translated into English. Initial familiarization with the data through repeated review of the transcripts marked the first analytical stage. The framework was developed on the basis of objectives and themes emerging from the respondents’ answers. The developed categories were re-evaluated and checked for reliability. As consequence, they could either stand for themselves or were subsumed under other categories, transforming them into subcategories. They were also compared to the categories from the B2C forms to control for credibility of the data. Themes and subthemes were illustrated by supporting quotes (Table 3). Interviews were coded in a database by two researchers to ensure reliability and consistency of the coding criteria (46). Discrepancies, when occurring, were openly discussed and resolved.

### Ethical approval

Informed consent for the participation in the B2C program was obtained as part of the clinic registration. The Malawi National Health Science Research Committee (NHSRC) approves the collection and retrospective analysis of routine data. Prior to the in-depth interviews, written informed consent was gained from all patients. Ethical approval was obtained from the Malawi NHSRC and the Ethical Research Board of the University Heidelberg/Germany.

## Results

### Patients’ characteristics

Between January 2008 and December 2009, 14,121 patients aged 15 years and above received ART at the Lighthouse. Within this period, 12.9% (*n*=1,826) of these patients were identified as LTFU prior to ascertaining their actual ART status by the B2C programme (Fig. 1). The B2C team identified 581 patients with complete stop of all prescribed antiretroviral drugs. A subset of these patients (*n*=347; 58.2% female, average age 35.1±11.3 years) returned to the clinic for continuation of therapy, here defined as patients with TI. Mean duration of ART among patients with TI(s) was 15±14.9 months (range 0–75 months), which is significantly below the mean for patients with uninterrupted therapy between 2008 and 2009 (mean 25.8±20.4; range 0–125 months; *p*<0.0001; adjusted for sex and age). Within the group of patients with a history of TI, 90.9% interrupted ART once, 7.8% twice and 1.25% three times within the study period. The mean duration of TI was 87±87 days (range 22–552) and was significantly longer in males (mean 103±105 days) compared to females (mean 75±68 days) (*p*=0.001, adjusted for age).

### B2C form

**Fig. 2:**
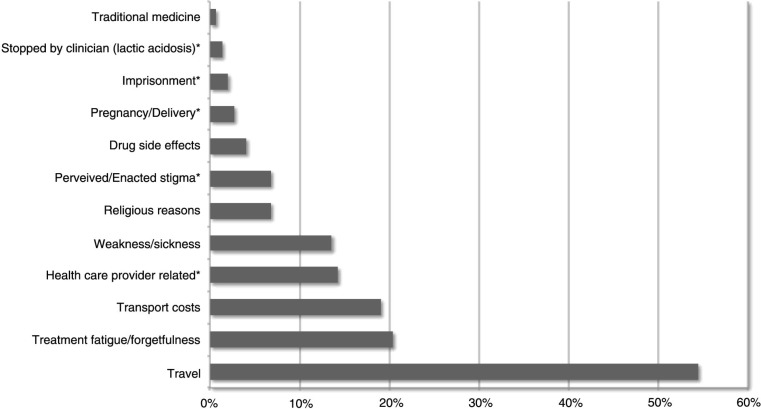
Reasons for treatment interruption. Relative frequency of reasons for treatment interruption as stated by patients in the B2C form *n*=147; multiple reasons per patient possible. Additional categories exceeding the scope of the pre-defined categories were derived from the comment field and are marked with a star: *. B2C: Back-to-Care.

In total, data from 147 patients with confirmed TI was entered. This subset did not differ significantly from the total number of patients with TI (*n*=347) with regard to their basic demographics (gender, age), duration of ART prior to TI, and duration of TI. The majority of forms were filled by the interviewer speaking with the patients themselves (83.7%, *n*=123/147), whereas if they could not be contacted the interview was conducted with the treatment supporter, the spouse or a close family member. In over a third of cases more than one reason for TI per patient was documented with the B2C form. The most commonly stated reason for TI was *travel* (54.4%; *n*=80/147; Fig. 2). According to additional comments *travel* was related to *travel* for *work* or *family issues*. The category *treatment fatigue/forgetfulness* was selected by the interviewer in 20.4% (*n*=30/147) of cases, but was only chosen six times as sole reason for TI and commonly associated with the category *travel*. Next, patients stated *transport costs* (19.0%, *n*=28/147) as reason for TI. Less frequently reported reasons were *weakness/sickness* (14%, *n*=20/147), *religious reasons* (7%, *n*=10/147) and *drug side effects* (4%, *n*=4/147). Only one patient stated TI due to use of *traditional medicine*. More than a quarter of the patients (28%, *n*=41/147) stated additional barriers to continuous care, which exceeded the scope of the pre-defined categories. The following reasons could be derived from additional free-text comments: *Health-care-provider-related reasons* were specified by 14% (*n*=21/147) of respondents. This category includes aspects such as *perceived rejection to receive ART re-fill* (i.e. lost health passport, site visits without appointment, admission to hospital), or a *disturbed relationship with the health care provider* through either perceived or enacted discriminatory behaviour. *Perceived or enabled stigma by family, friends or the employer* (7%; *n*=10/147) was also an obstacle to remain in care. Some women (3%; *n*=4/147) interrupted therapy due to conditions related to their *pregnancy or delivery*. Rare reasons were *imprisonment* (2%; *n*=3/147) and *stopped ART by a clinician* due to lactate acidosis with consecutive LTFU (1%; *n*=2/147).

### In-depth interviews

Fifteen females and eleven males were interviewed. The median time of interview was 18 min. Demographics such as age, sex, marital status, occupation and duration of ART prior to TI were recorded (Table 1).

**Table 1:** Respondents’ characteristics: In-depth interview (*n*=26)

	Female (*n*=15)	Male (*n*=11)	Total (*n*=26)
Age			
Median	26	32	30
15–19	3	0	3
20–29	7	3	10
30–39	3	5	8
40–50	2	3	5
Marital status			
Single	1	2	3
Married	9	8	17
Divorced/separated	3	1	4
Widow	2	0	2
Occupation			
Small scale business	6	4	10
Housewife	2	0	2
House servant/cook	3	0	3
Teacher	2	0	2
Student	1	0	1
(Truck) -driver	0	2	2
Gardener/farmer	1	2	3
Prisoner	0	1	1
Mechanic	0	1	1
Minibus caller	0	1	1
Duration on ART (months)^a^			
Only starter pack^b^	3	0	3
2–6	6	6	12
7–12	1	2	3
13–24	1	2	3
25–36	2	1	3
>36	2	0	2

ART: Antiretroviral therapy.

a Duration on ART prior to interruption period in months.

b Patients initiated on ART receive a starter pack (14 day supply of stavudine and lamivudine).

#### Factors known to support drug adherence and retention in care

##### Basic knowledge about HIV/AIDS and ART

Generally respondents had sufficient knowledge about HIV/AIDS, the effect of antiretroviral drugs, the importance of adherence, as well as possible consequences of TI (Table 2). All respondents confirmed that the health care providers gave information about ART at the time of therapy initiation. Almost all respondents were able to differentiate between HIV and the development of AIDS. Similarly, almost all respondents were aware of ARVs’ mode of effect on HIV and the recovery of the immune system as well as the importance of regular drug intake as prescribed. All respondents knew about ART as a lifelong therapy (for illustrative quotes see Table 2).

**Table 2:** Factors supporting with drug adherence and retention in care in patients with documented treatment interruption (in-depth interviews; *n*=26)

Basic knowledge about HIV/AIDS and ART	Illustrative quotes
The patient was able to …	
… differentiate between HIV and AIDS	*HIV is a virus that causes AIDS, while AIDS is the accumulation of different diseases caused when body immunity is low*. (Female, 29 years, 37 months on ART)
… describe the effect of ARVs	*It is a type of medication that boosts one's immune system and it is taken in every day of one's life, when one is HIV positive. Again ARVs help to prevent opportunistic infections*. (Male, 33 years, 28 months on ART)
… understand that ART is a lifelong therapy	*Because the virus will not be healed but will remain in the body the rest of life. It does not mean that when we take the drugs, the HIV/AIDS will go, no. They are not like cough treatment where we get healed, so we need to take them the rest of our life*. (Female, 17 years, on starter pack)
… describe the possible consequences of an interruption of ART	*The importance is that when you adhere to treatment the virus cannot resist drugs and that we will have healthy life*. (Female, 28 years, 22 months on ART)
Social support systems	Illustrative quotes
Disclosure to …	
… relatives.	*Disclosure to relatives:* *I told them to know so that I may get proper care and help when I am sick, and also to know where to seek care, better a government facility than a private clinic*. (Female, 26 year)
… close friends.	*Disclosure to spouse:* *So that he may as well go for HTC [HIV testing and counselling]*. (Female, 33 years)
… employer, church leader and/or neighbour.	*Disclosure to employer:* *So that he should not be surprised whenever I make an excuse to be absent from work to go to the hospital as it has happened today*. (Male, 32 years)
Support group	*We encourage each other and care for people who are very ill, that can't work on their own*. (Female, 29 years, married)
Support through church/religion	*They normally talk of how best an HIV patient can live as well as encouraging people to adhere to treatment*. (Female, 39 years)
Treatment supporter/guardian*	*I was advised to come for another counselling session with a guardian at a given date but a problem was which guardian [treatment supporter] I could bring since my wife is away, my brother is a busy person*. (Male, 33 years)

AIDS: acquired immunodeficiency syndrome; ART: antiretroviral therapy; HIV: human immunodeficiency virus.

* All respondents had a documented treatment supporter; however, some patients stated challenges as shown in the illustrative quote.

##### Social support systems

All respondents reported that they disclosed their HIV and treatment status to at least one other person. The choice of whom to disclose to was mainly based on trust and belief that the person would respect and maintain confidentiality. All but one of the 17 married respondents disclosed to their spouses. Most participants disclosed to relatives and close friends. A few participants had disclosed to their employers, church leaders and/or neighbours. The main reason for disclosing was to ensure that in case of sickness a trusted person could provide and seek care. In addition, patients disclosed to their spouse in order to encourage them to go for HIV testing and counselling. Some participants attended support groups. Most patients attending support groups were female. More than two thirds of the respondents reported that their church supports HIV testing and counselling as well as ART.

As a prerequisite for being initiated on ART in Malawi and at the Lighthouse, patients are requested to name a treatment supporter (‘guardian’) who is willing to support him/her adhering to therapy. These guardians are allowed to collect drugs instead of the patient, that is, in case of illness. Some interviewees stated that disclosing to only one person was challenging in case the trusted person died or travelled. As a result, patients struggled to identify another person who could step in as a guardian (for illustrative quotes see Table 2).

#### Reason for TI

Based on the in-depth interview and information derived from comments in the B2C form, the category *travel* was differentiated into the subcategories *work-* and *family-related travel*. Notably, men who travelled far distances for a job obligation mainly stated *travel for work*. Whereas women mostly stated *family-related travel*, for example, they took care of a relative and had no means to return home. Second, patients described *health-care-provider-related conditions* like loss of health passport, missing transfer letters and perceived or enacted discriminatory behaviour of health care workers as the explanation for delayed return to care. Another important reason was financial limitations such as *transport costs*. Furthermore, *self-reported health conditions* including *adverse effects, sickness/weakness, pregnancy* and *treatment fatigue* were also mentioned in in-depth interviews as drivers of TI (Table 3).

**Table 3:** Reasons for treatment interruption (in-depth interviews; *n*=26)

Categories	Definition	Illustrative quotes
Travel (*n*=16)		
Family related	Patient was travelling far in order to support family members. The distance to the clinic caused the treatment interruption.	*I went to a funeral in Balaka, but failed to come back on time because I didn't have transport money*. (Female, 22 years)
Work related	Patient was travelling far because of any kind of work, a job opportunity or assignment. The distance to the clinic caused the treatment interruption.	*Since I am a labourer, I went to Dzalanyama to collect firewood, unfortunately had a car breakdown, slept there and did not carry my medicine*. (Male, 44 years)
Financial barriers (*n*=7)		
Transport cost	The patient could not afford the transport to the health centre, because of the distance and/or the lack of money.	*Sometimes I even lack transport to come here from Chinsapo to collect my medication*. (Female, 23 years)
Own health related (*n*= 6)		
Adverse drug effects	The patient suffered from strong adverse effects of ART. He/she stopped treatment in order to improve his/her well-being.	*I was having rashes, dizziness, stomach-ache and swollen legs* (Female, 26 years)
Weakness/Sickness	The patient suffered from any symptoms not necessarily related to ART. He/she stopped because of illness.	*I stopped because I was sick; had terrible headache and coughing* (Female, 24 years)
Pregnancy/delivery	The patient was pregnant and or delivered a baby when she was supposed to collect drugs from LH/MPC. Her special condition detained her from collecting the drugs.	*That time I had just delivered this baby so had no strength to walk to here*. (Female, 33 years)
Health provider related (*n*=8)		
Perceived rejection to receive ART re-fill	The patient felt/was told not to fulfil the requirements to collect ART at the centre. He/she therefore avoided going to the health centre.	*I lost my health passport book through robbery. […] I thought that I could not be helped without this book*. (Female, 42 years)
Disturbed relationship to health care provider	The patient experienced/perceived difficulties collecting new drugs from the health centre. He/she therefore avoided going to health centre.	*I was afraid I could be shouted at by the Doctor* (Female, 22 years)
General adherence (*n*=1)		
Treatment fatigue	The patient was overwhelmed by the challenge to adhere to ART. He/she gave up and stopped therapy.	*Laziness, and sometimes I take the medication without food, which raises fears in me*. (Female, 23 years)

## Discussion

We aimed at understanding reasons for TI in patients on ART at the Lighthouse Clinic in Lilongwe, Malawi. We evaluated a specific group of patients who temporally interrupted ART, meaning these patients stopped ART for a certain period of time but ultimately returned to care to resume therapy. Evaluating their key characteristics, factors supporting adherence and retention in care, as well as individual circumstances leading to TI can inform future adherence counselling: The provision of problem-solving strategies targeting common barriers to care is required to adequately support patients on a lifelong therapy.

Differentiation between non-adherence and TI as separate treatment outcomes (47), as done in this study, is of growing importance. Kranzer et al. presented a systematic review of studies from developed and developing countries specifically reporting TI as outcome and evaluating frequency, reasons, risk factors, and consequences of TI (31). However, only few of these studies investigated reasons for TI for the setting of sub-Saharan African countries especially for countries with ART available free of charge.

Treatment guidelines at the Lighthouse, including ART regimens and treatment monitoring, can be considered representative for Malawi (48). However, the study site's capacity regarding the number of patients treated, quality management as well as monitoring and evaluation efforts exceeds the average ART site in Malawi. Additional features, such as the early active follow-up (B2C) programme, aim to improve retention in care. Efforts to retain patients appear to be successful since only 4.1% of the overall treatment cohort stopped/interrupted ART, which compares favourably to other settings (10). Remarkably, more than half (59.7%) of these patients returned to the clinic and resumed therapy. Furthermore, most programmes do not trace patients LTFU and therefore cannot further distinguish their ART status as done in this study setting to specifically target patients with confirmed TI.

We evaluated key characteristics of patients with confirmed TI in comparison with patients who regularly attended their scheduled clinical visits: The mean duration of ART was significantly shorter in patients interrupting ART compared to patients who continuously attended clinic visits; and duration of TI was significantly longer in men when compared to women. This is consistent with previous findings where stop of ART was associated with male gender and time since ART initiation – particularly during the first 6 months; and ART resumption was more likely in women (30, 49). Retention in care could be further improved by providing adherence counselling specifically targeting patients in the first months after ART initiation and services tailored for male patients. Amongst other recommendations, Kranzer at al. proposed late clinic hours providing care after working hours, special support groups for men and adherence counselling specifically dealing with the needs of male patients (49).

In-depth interviews revealed a sufficient knowledge about HIV/AIDS, the effect of antiretroviral drugs and possible consequences of TI in most of the interviewed patients. This reflects the emphasis the Lighthouse Clinic places on treatment readiness assessments, adherence counselling and regular patient education sessions. All patients disclosed their HIV status to at least one person, most frequently their spouse. The idea of disclosure is to reveal one's HIV status to a trusted person who can maintain confidentiality and offers support if needed. Patients who have not disclosed their HIV status to their partners are more likely to miss doses than other patients (50–52). In our study, the motivation behind disclosure appeared to be driven by the need for assigning or having a treatment supporter. Treatment supporters have the task to remind the patient of scheduled dosing times and clinic appointments (50). Having a treatment supporter is part of the ART readiness assessment in Malawi. When a treatment supporter travelled or died, some patients struggled to identify another person to step in to collect drugs on their behalf. Consequently, the positive idea of a treatment supporter might turn into an obstacle, because patients felt they were no longer eligible for ART continuation. Rachlis et al. made a similar observation in another patient cohort in Malawi where lack of a treatment supporter was incorrectly perceived as ineligibility for ART as part of hospital policies (53).

Considering that most interviewees showed sufficient knowledge about HIV/AIDS and ART and had treatment supporters at their side, the study explored what other reasons caused them to interrupt treatment. Previous research from Malawi identified high transport costs, perceived stigma, dissatisfaction with care, improved health, poor understanding of the disease and treatment as well as drug side effects as main causes for TI (54–56). Evaluating the pre-defined categories of the B2C form, the most commonly stated reason for TI was *travel* followed by *treatment fatigue/forgetfulness* and *transport costs*. The analysis of free-text comments revealed additional barriers to continuous care exceeding the scope of the pre-defined categories such as *health-care-provider-related* reasons for TI. The selection of multiple pre-defined categories as well as the variety of additional reasons for TI derived from free-text comments stressed the need for in-depth analysis. We therefore conducted a qualitative analysis of the free-text comments and conducted a number of purposively sampled in-depth interviews to gain a deeper understanding of pre-defined and newly derived reasons for TI.

The pre-defined category *travel* was further differentiated into *travel* related to *work obligations* or *family issues*, such as taking care of a relative or attending a funeral. These causes reflect both economic and strong personal factors, which apparently outweighed patients’ knowledge and awareness of the possible consequences of TI and were given priority over keeping an appointment. Despite these personal circumstances most of these patients were generally willing to continue ART. The provision of additional drug supplies on short notice as well as the transient transfer to other ART facilities could be part of a more flexible ART programme responding to the patients’ individual circumstances.

The category *treatment fatigue* included *forgetfulness* or *feelings of being overwhelmed by the challenge to adhere to ART*. New approaches try to use mobile phones and text messages to remind patients of their next appointment, a technology which can also be used for real-time measure of adherence among patients receiving ART (57). Studies using mobile devices in Ghana and Kenya demonstrated improved patient attendance and day-to-day drug adherence (58, 59). However, reasons for TI as found in this study go beyond general forgetfulness and our data suggests that ‘simple’ technical reminders might be useful but not necessarily sufficient in all cases. In this study, *treatment fatigue* was only stated six times as the sole reason and commonly associated with the category *travel*. This phenomenon might therefore be a result of an interviewer bias, as the B2C team summarized the patient's statements under the pre-defined categories. This is also supported by the findings of the free-text comments and in-depth interviews revealing more specific reasons for TI.

Another main reason for TI was reported to be *transport costs*, which hindered patients from coming to the clinic. *Transport costs* constitute a barrier to care, as they often compete with other costly demands such as food, housing or school fees, thereby compromising access to care (34). This in turn may result in the interruption of treatment. According to Boyer et al. (47) financial factors appear as main drivers of TI (47). Meanwhile ART in Malawi is provided at 655 sites and free of charge, which is thought to reduce transport costs and increase accessibility to ART (60). However, this advantage may be partially counteracted by increased travel cost following inflation (61).

Further, the category of *health-care-provider-related* reasons, such as *perceived ineligibility for ART*, for example, the lack of a treatment supporter or a health passport or a *disturbed provider–patient relationship* was reported in free-text comments of the B2C form. This finding underlines the importance of the interaction between the individual patient and the health care provider. Misleading and rigid hospital policies should be avoided and the health care provider needs to be perceived as a partner in care reducing logistical barriers. The acknowledgement of the patient's perception to be stigmatized (53) and the prevention of discriminatory behaviour of the health personnel is also crucial for the health care provider – patient relationship.

However, it should be noted *that treatment fatigue, transport costs* and *health-care-provider-related reasons* combined were less frequently reported than *travel* alone, underlining its relevance and stressing the need to address the issue.

Our findings may therefore result in practical implications for patient management and adherence support strategies. Rather than ‘simple’ technical reminders such as text messages, more flexibility in the drug re-fill schedule and counselling addressing the identified reasons for TI providing problem-solving strategies are needed, which have already been partially introduced at the Lighthouse as a result of this study.

### Limitations

Our study had several limitations. First, only patients with TI resuming therapy were included. Patients not returning to care could not be interviewed and may have other reasons for stopping ART completely. It needs to be emphasized that at the Lighthouse the majority of patients resume therapy after a period of interruption and we were particularly interested in their reason for TI. Our findings confirmed that these patients interrupted ART for temporary obstacles and most of them never intended to ultimately stop ART.

Second, ART counsellors filled B2C forms and the evaluation of additional comments was done retrospectively. Also, it cannot be fully determined to what degree respondents replied truthfully or whether answers were subject to desirability bias. Finally, only subsets of patients with TI were included for the analysis of B2C forms and only few patients were further selected for in-depth interviews. Although basic characteristics showed no significant differences between patients with TI and the subset of enrolled patients for the analysis of B2C forms, and in-depth interviews were continued until a point of saturation was reached, this approach might be subject to a selection bias.

## Conclusion

Improved retention in care and adherence to therapy must be considered as two of the main pillars of national ART programme success. Both retention and adherence have the potential to effectively reduce subsequent treatment failure. Addressing the challenges of supporting patients on lifelong therapy, strategies must be developed that prevent or reduce discontinuation of ART. A profound understanding of reasons for TI is the basis for interventions such as needs-adjusted counselling, providing problem-solving strategies specifically targeting common adherence challenges like travel or health-system-related barriers.
